# Regenerative Medicine: A Review of the Evolution of Autologous Chondrocyte Implantation (ACI) Therapy

**DOI:** 10.3390/bioengineering6010022

**Published:** 2019-03-13

**Authors:** Rebecca L Davies, Nicola J Kuiper

**Affiliations:** 1 Institute for Science & Technology in Medicine (ISTM), Keele University, Newcastle ST5 5BG, UK; r.l.davies@keele.ac.uk; 2 Arthritis Research Center, The Robert Jones & Agnes Hunt (RJAH) Orthopaedic Hospital, Oswestry SY10 7AG, UK

**Keywords:** articular cartilage, regenerative medicine, chondron, extracellular matrix, autologous chondrocyte implantation (ACI), cell therapy

## Abstract

Articular cartilage is composed of chondrons within a territorial matrix surrounded by a highly organized extracellular matrix comprising collagen II fibrils, proteoglycans, glycosaminoglycans, and non-collagenous proteins. Damaged articular cartilage has a limited potential for healing and untreated defects often progress to osteoarthritis. High hopes have been pinned on regenerative medicine strategies to meet the challenge of preventing progress to late osteoarthritis. One such strategy, autologous chondrocyte implantation (ACI), was first reported in 1994 as a treatment for deep focal articular cartilage defects. ACI has since evolved to become a worldwide well-established surgical technique. For ACI, chondrocytes are harvested from the lesser weight bearing edge of the joint by arthroscopy, their numbers expanded in monolayer culture for at least four weeks, and then re-implanted in the damaged region under a natural or synthetic membrane via an open joint procedure. We consider the evolution of ACI to become an established cell therapy, its current limitations, and on-going strategies to improve its efficacy. The most promising developments involving cells and natural or synthetic biomaterials will be highlighted.

## 1. The Complexity of Adult Articular Cartilage

**Figure 1:**
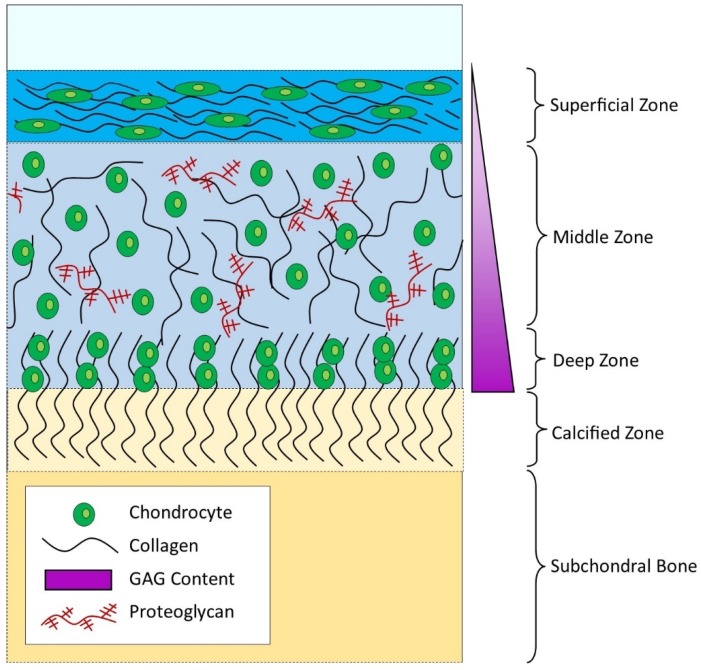
The structure of articular cartilage and the underlying subchondral bone. From articular cartilage surface to the bone there are four zones—superficial, middle, deep, and calcified. Within the zones, there are differences in the orientation of the collagen fibers, the arrangement of the chondrocytes, and the distribution of proteoglycans and their associated glycosaminoglycans (GAGs).

Articular cartilage acts as a cellular cushion, ensuring that our joints withstand the physical and mechanical demands of everyday life. Within articular cartilage, each chondrocyte is surrounded by a 2–4 µm thick collagen VI-rich pericellular matrix (PCM) forming a chondron [1,2]. The PCM allows chondrocytes to adapt to the micromechanical pressures during load bearing [3,4,5]. In turn, chondrons are surrounded by a territorial matrix and a highly organized extracellular matrix (ECM) comprising collagens, proteoglycans (mainly aggrecan), glycosaminoglycans (GAGs), and non-collagenous proteins [6,7]. With increasing depth from the articular surface, there are four architectural zones (Figure 1) with striking variations in both chondrocytes and their surrounding ECM [8]. In brief, collagen fibers in the superficial zone are densely packed and oriented parallel to the articular surface, in the middle zone they have a random arrangement, and in the deep/calcified zones they are oriented perpendicularly to the articular surface. The amounts of proteoglycans and GAGs vary with depth; their concentrations greatest in the middle to deep zones [9,10].

## 2. Articular Cartilage Injury as a Risk Factor for Osteoarthritis

Despite the high demands of this tissue, self-repair is highly inefficient due to the lack of blood supply and the sparse population of immobile chondrocytes. Many factors are known to increase the risk of getting osteoarthritis (OA), such as a defect of cartilage or an injury. Cartilage defects can be clinically defined as partial thickness chondral, full thickness chondral, or osteochondral [11]. Cartilage injury can lead to cell death, which is detrimental to tissue homeostasis and has the potential to progress towards OA where the loss of articular cartilage is a major outcome [12,13]. Early OA is characterized by chondrocyte proliferation, chondrocyte clustering, and an increased synthesis of irregular cartilage ECM components. Late OA is characterized by excessive catabolic activity, which leads to an imbalance in cartilage homeostasis, resulting in breakdown and loss of aggrecan. These catabolic events are largely mediated by pro-inflammatory cytokines and mediators such as matrix metalloproteinases.

## 3. The Surgical Strategies for Treating Cartilage Injury

Several surgical approaches exist to repair damaged articular cartilage (Table 1) with variable outcomes due to factors associated with the individual’s demographics, the injury, and any previous surgical procedures [14,15]. When choosing the surgical approach, it is important for the surgeon to consider the individual needs of the patient, with respect to the joint that is affected and the patient’s hobbies and lifestyle. Consequently, what works for one patient, may lead to a different outcome in another.

## 4. The 20-Year Evolution of ACI from Pilot Study to Long-Standing Surgical Technique

**Figure 2:**
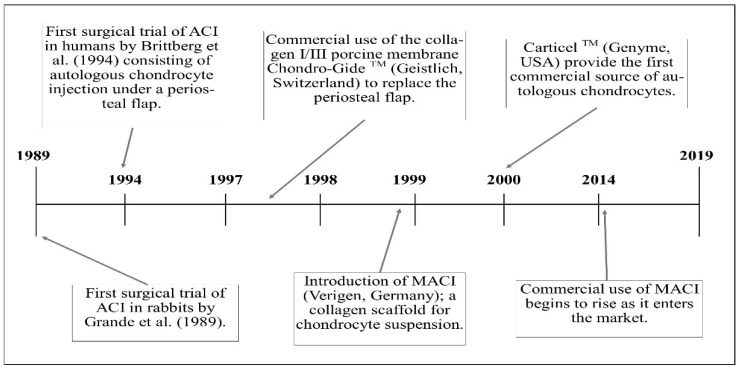
A timeline consisting of the significant events allowing the evolution of autologous chondrocyte implantation (ACI) to its current form. MACI—matrix-autologous chondrocyte implantation. Adapted from [23,25,26,27,28].

Autologous chondrocyte implantation (ACI) was first used in humans in 1987 with the first pilot study published in 1994 [23]. By 2010, over 35,000 patients had been treated worldwide [24]. Currently, ACI is the only cell therapy available in the UK on the National Health Service (NHS) for the treatment of cartilage defects. Figure 2 shows the timeline and major milestones during the evolution of ACI, which will be described in the narrative to follow.

### 4.1. First Generation ACI

**Figure 3:**
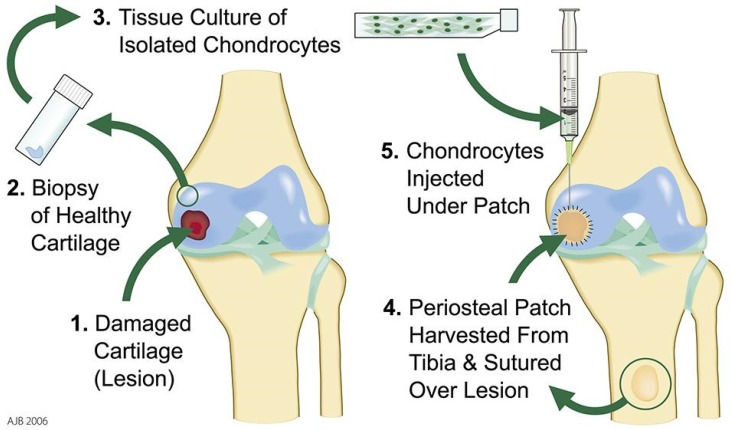
An overview of first-generation autologous chondrocyte implantation (ACI) in which a biopsy of healthy cartilage was removed arthroscopically. Chondrocytes were expanded in culture. An arthrotomy enabled the surgeon to re-inject the cell suspension under an autologous periosteal patch. Image accredited to Mr. Andrew Biggs, Robert Jones and Agnes Hunt Orthopedic Hospital, Oswestry, Shropshire, UK.

The first generation of ACI involved re-implanting monolayer expanded autologous chondrocytes into the damaged region under a natural or synthetic membrane via an open joint procedure (Figure 3). Surgeons reported that this was technically challenging due to complications associated with the periosteum [29]. During surgery, periosteal shrinkage resulted in the need for additional periosteum harvesting with some patients reporting post-operative pain at the harvest site. During surgery, tearing of the fragile periosteum and the complexity of suturing were demanding for the surgeon. Our center and others showed a high percentage of good to excellent clinical results with a failure rate of around 16% [30,31]. All post-operative failures occurred within the first two years. Failure was most often due to periosteal hypertrophy, in which overgrowth of the periosteum typically required surgical intervention to shave the graft, or delamination of the periosteal tissue [32]. Commercial ventures developed alongside ACI evolution, with Genzyme, USA offering Carticel™ as the first service to expand autologous chondrocytes from harvested biopsies [33]. The cartilage cell therapy journey had begun in earnest.

Initially there was a lack of quantitative and qualitative biochemical data regarding the nature of ACI repair tissue due to the reluctance to disturb the repair site. After establishing our Good Manufacturing Practice (GMP) cell therapy laboratory for musculoskeletal tissues, the first in the UK, we were in a unique position of being able to access the repair tissue. We were not only able to report on early findings but also to follow up our patients over an extended time. For example, we reported that at one year, magnetic resonance imaging (MRI) of the operated joint and histology of the repair tissue could be used to monitor early success in 2003 [34] as well as predicting the long-term success for an even larger cohort 17 years later in 2018 [35]. With histological, immunohistochemical and detailed glycan analysis approaches we were able to build a picture of key ECM markers such as collagen [36] and GAGs within the repair tissue [37,38]. In turn, our knowledge contributed to the fast-moving wider cartilage repair field.

### 4.2. Second Generation ACI

Issues with the native periosteum were rapidly resolved as ACI moved to the second generation. Geistlich, Switzerland produced Chondro-Gide™, a porcine collagen I and III membrane, to replace the periosteum. Preliminary studies [27] with 31 patients gave satisfactory outcomes although re-operation was still required to deal with a smaller number of cases of delamination, graft failure, or the inability of the regenerated tissue to integrate with the surrounding native cartilage [39]. Despite this, long-term follow-up [40,41] provided evidence that Chondro-Gide™ alleviated pain and swelling to increase the level of knee functionality and appeared to result in a better quality of repair tissue.

### 4.3. Third Generation ACI

Third generation ACI, consisting of suspending expanded chondrocytes in a hydrated scaffold, was commercially developed by Verigen, Germany and is more recently produced and marketed by Vericel, USA. It is termed matrix-autologous chondrocyte implantation (MACI™). It has several advantages which include removing the need for a native or synthetic periosteal patch, better control of cell distribution throughout the defect, and the potential to manage more extensive osteochondral defects. With respect to the latter, in clinical trials, MACI has typically been used to tackle lesion sizes ranging from 2.3 ± 1 to 11.8 ± 8 cm^2^. Several independent studies exploring MACI have shown significant improvement with respect to clinical outcomes [25,42,43] with many patients reporting good knee functionality with reduced pain and swelling. The reported failure rate is 10.7% at seven years [44] compared to 33% failure rate with first generation ACI [32].

## 5. What Are the Key Limitations of ACI in Its Current Form?

Isolated autologous articular chondrocytes are a long-established cell source for ACI, but they might not be the ideal form of chondrocyte to produce cartilage ECM. The optimum number of chondrocytes required to fill a cartilage defect is still not reported. What is clear is that any increase in chondrocyte number from a small cartilage biopsy requires in vitro expansion. Chondrocyte de-differentiation [45], during in vitro monolayer expansion, and the resulting decreased capacity of re-implanted chondrocytes to regenerate hyaline articular cartilage, remain key limitations of ACI. In articular cartilage, each chondrocyte is surrounded by a 2–4 µm thick collagen VI-rich PCM forming a chondron. Freshly extracted chondrons form a more cartilage-like ECM than chondrocytes [46,47], and their surrounding PCM is thought to maintain chondrocyte phenotype. One possibility is to isolate adult chondrons but with each cartilage harvest for ACI containing only a small number of chondrocytes (2000–4000 chondrocytes per mg) this will only yield a small number of chondrons (35–40% less—unpublished observation). These chondron numbers alone are too small to affect a cartilage repair within a defect. However, there is a recent prospective, randomized, controlled trial [48] using different doses of spheroids of neocartilage composed of expanded autologous chondrocytes and their associated PCM (Spherox or Chondrosphere™; a form of ACI for repair of 4–10 cm^2^ defects). The clinical outcomes have yet to be published.

ACI in its current form is not promoted as a treatment for OA but probably the ultimate objective of cell therapy is to target a synovial joint with late OA. When applied to OA, the ambition of a cell-based therapy would be to bring about a permanent repair without the need for further long-term surgery, for example a joint replacement. With all the above in mind, we will highlight the on-going research surrounding novel tissue sources and cell types which are being developed and utilized for cartilage cell therapy.

## 6. Where Are We with Respect to Tissue Sources and Cell Types for Cartilage Cell Therapy?

### 6.1. Adult Nasal Chondrocytes

In early investigations of the chondrogenic capacity of adult chondrocytes [49], cells sourced from adult nasal cartilage had a greater capacity for collagen II and GAG production than articular chondrocytes over long-term culture. More recently, adult nasal chondrocytes have been shown to have a higher proliferative potential when compared to adult knee chondrocytes [50]. In the first-in-human clinical trial, Mumme et al. [51] showed that cartilage repair occurred with collagen and GAG production on par with articular chondrocytes. Together these findings indicate that nasal chondrocytes might well contribute to a high level of repair, producing hyaline rather than fibrocartilage. Moreover, this cell source is advantageous since it does not require interference with already compromised cartilage to enable repair.

### 6.2. Human Embryonic Stem Cells (hESCs)

hESCs can differentiate into multiple cell types of the three embryonic germ layers. First established in 1998, hESCs have opened an entirely new avenue for regenerative medicine [52]. Protocols have been established to direct hESCs towards a chondrogenic fate for cartilage regeneration [53,54]. Chondroprogenitor cells have been encapsulated in a fibrin gel and implanted into an osteochondral defect in a rat model. At 12 weeks post-implantation, the defect sites within the models were filled with ECM, which was histologically confirmed to be hyaline-like cartilage. The cartilage was also proved to be derived from the implanted cells, thanks to cellular tracking of their lineages. This process has been replicated in human explanted OA knees, where similar evidence has confirmed the formation of hyaline-like cartilage [55]. The regenerated cartilage has reproduced the complex ECM organization of native cartilage, including the morphology of cells and distinct protein composition of each of the architectural zones. Interestingly, proteoglycans and their associated GAGs were concentrated in the implant area suggesting a promotion of chondrogenesis. Despite the promise of hESCs, we currently lack the knowledge that establishes their superiority over autologous chondrocytes. The evidence presented by Olee et al. [55] showed that hESCs have poor expression of chondrogenic factors in comparison to adult chondrocytes. This could hint at an incomplete differentiation protocol and further work is ongoing [56].

### 6.3. Inducible Pluripotent Stem Cells (iPSCs)

iPSCs are a source of pluripotent cells derived from fully differentiated adult cells [57]. They are derived by modifying the expression of unique transcription factors to restore their pluripotency. To date, iPSC-derived chondrocytes have been shown to embody the benefits of juvenile chondrocytes [58]. This includes an increased proliferative potential, with doubling times less than 10 hours compared to adult chondrocytes. This is suspected to be due to differential gene expression, more specifically the up-regulation of CD24 molecule, which has been shown to convey both proliferative potential and immunological resistance for iPSC-derived chondrocytes. This means that iPSCs have the potential to be generated faster in vitro, thereby increasing turnover of patients through a decreased culture time. Immunological resistance suggests the promotion of graft integrity and the formation of cartilage in vivo. By contrast, analysis of gene profiling has identified an up-regulation in genes associated with stress response and DNA damage, hinting at an increased risk of genetic instability [59]. This conveys the increased likelihood of tumorigenesis because of increased exposure to multiple stress events in vivo. Hence, quality control is essential when using these cells to avoid unwanted and dangerous complications.

Today, iPSCs are being used to generate cartilage to repair knee defects in animal models [60]. Upon confirmation of a chondrogenic fate, results exhibited an impressive level of regeneration upon histological evaluation, perhaps even superior to that shown by hESCs. Unfortunately, these were limited in comparison to healthy cartilage and have yet to achieve optimal regeneration. Even so, the use of iPSCs were deemed safe since there was no teratoma formation. Therefore, as with hESCs, we can conclude that they have potential for use in cartilage cell therapies.

### 6.4. Bone Marrow-Derived Mesenchymal Stem Cells (BM-MSCs)

Mesenchymal stem cells sourced from bone marrow (BM-MSCs) can differentiate into multiple lineages including osteoblasts, adipocytes, and chondrocytes themselves [61] making them an ideal cell source for cartilage repair and regeneration. The safety of BM-MSCs has been well established [62,63] since Watkitani, a true pioneer within this field, performed the first in-patient trial in 1988 by implanting autologous BM-MSCs to repair articular cartilage in a ground-breaking collaboration with Arnie Caplan [64,65]. To date, there is an equal weight of evidence suggesting that BM-MSCs with or without other cell types are suitable alternatives to ACI. This is evidenced, for example, by the observational cohort study by [66], which reported that BM-MSCs in cartilage repair are as effective as chondrocytes. Long-term follow-up is clearly important to establish the safety of BM-MSC therapy. Several other centers, including our own, have run or are currently running clinical trials with long-term follow-up using autologous or allogenic BM-MSCs in different approaches. Akgun et al. [67] compared autologous BM-MSCs with chondrocytes and reported better healing with BM-MSCs. Our center is concluding the first clinical trial (ASCOT ISRCTN98997175) in the UK to compare autologous BM-MSCs with chondrocytes with results to be reported soon. Autologous BM-MSCs in an approach that is similar to MACI have resulted in impressive clinical outcomes [68]. Using a goat model, a Netherlands-based team first showed that human chondrons produce more cartilage ECM than human chondrocytes, when cultured with BM-MSCs [69]. Their work was translated to the clinic (IMPACT clinical trial NCT02037204) to assess the safety and efficacy of allogenic BM-MSCs combined with autologous chondrons [70,71].

Initially BM-MSCs were sourced for cartilage cell therapy because of their proven ability to differentiate into chondrocytes and as a positive enhancement to existing ACI. Over the last decade, their differentiation has been found to be modulated by growth factors, mechanical stimulation, co-culture with chondrocytes or chondrons, and interactions with the ECM. Several studies have shown that co-culture of chondrocytes or chondrons with BM-MSCs resulted in superior ECM production compared to cultures containing only chondrocytes or chondrons alone [72,73,74]. Evidence has revealed that BM-MSCs convey these actions via trophic factors [75,76] secreted in exosomes, which were first described by Lai et al. [77]. To date, weekly intra-articular administration of human embryonic-MSC exosomes can promote cartilage regeneration in animal models [78]. There is evidence to show that that human mesenchymal stem cell (MSC) exosomes do not have major histocompatibility complex (MHC) class I or II proteins [77,79], which is encouraging when we consider their application for allogeneic cartilage repair strategies.

### 6.5. Adipose-Derived Stem Cells (hADSCs)

Although BM-MSCs have been the focus for cartilage repair, hADSCs might be a more suitable alternative for cartilage cell therapy. By comparison to BM-MSCs, they are easier to harvest, found in higher frequency, and stable in long-term culture [80]. Garcia et al. [81] compared the in vitro chondrogenic potential of MSCs derived from bone marrow, infra-patellar fat pad, and subcutaneous fat from matched patient samples. They reported that hADSCs’ chondrogenic potency was closely matched to that of chondrocytes and BM-MSCs. hADSCs harvested from the infra-patellar fat pad of patients with OA can produce hyaline-like cartilage when cultured in 3D gels [82], and there are some promising pilot studies showing that they also work in animals [83,84].

### 6.6. Allogenic Chondrocytes

In addition to these cell types, there is the emerging treatment method of allogeneic transplantation. Infant cartilage tissue has been obtained from polydactyly surgical waste offering a completely different cell source [85]. INVOSSA™ (Kolon TissueGene, Korea), currently in phase III clinical trials, is an allogenic cell therapy which uses chondrocytes isolated from polydactyl infants for the treatment of OA knees. Kolon claims that one injection of INVOSSA™ could provide the patients with over two years of productive and pain-free mobility, without the immediate need for surgery. It is an exciting development within the cell therapy field.

## 7. Where Are We with Respect to Natural and Synthetic Scaffolds for Cartilage Cell Therapy?

Following the promising clinical results of MACI, many unique compositions of biological scaffolds are available, with collagen currently dominating due to its natural presence in the ECM [8]. Recent work has shown that collagen I constructs may have immunomodulatory properties, as chondrocytes seeded onto collagen I hydrogels produced a lower immune response [86]. This conveys an advantage to the tissue, because it decreases the likelihood of immune rejection of the transplant. NOVOCART® 3D (TETEC Tissue Engineering Technologies, Reutlingen, Germany), a collagen I bilayer, which is currently in phase III trials, has shown significant improvement in clinical scores [87]. Another center has reported a high occurrence of hypertrophy [88] in 26.8% (11/41) of patients evaluated up to two years. With this setback, 19.4% of patients had to undergo further surgical intervention compared with only 10.7% who had undergone MACI [44].

Another popular consideration for scaffold material is hyaluronic acid (HA), which has a strong evidence base for supporting chondrocyte growth and ECM production. HA has been investigated in its many forms ranging from simple hydrogels to more complex benzyl esters with mixed success. With the most well-known HA-based scaffold, HYAFF-11 (Fidia Advanced Biopolymers, Italy), being utilized in a MACI™ approach as a carrier for autologous chondrocytes [89]. In recent years, HA has been shown to support the chondrogenesis of both MSCs and chondrocytes, as well as promote proliferation in vitro [90]. HA can support the maintenance of OA chondrocyte phenotype whilst increasing the amount of GAG production per cell [91]. Additionally, research by Zhu et al. [90] has proven the success of HA-based scaffolds in combination with collagen I or other GAGs. These modifications increased differentiation of MSCs towards a chondrogenic phenotype and decreased expression of hypertrophy-related genes with results confirmed both in vitro and in vivo using nude mice models. Amann et al. [68] also explored multiple parameters, including the variation of HA concentration in established collagen I-based scaffolds, which resulted in increased GAG production. Use of these scaffolds in co-culture has been shown to benefit chondrogenesis. All the evidence hints that the future of cartilage repair may not be the use of alternate cell sources, but a multicellular approach. Despite being highly complex due to specific interactions, this is more typical of a cellular system, which better promotes regenerative behavior.

Scaffolds will continue to evolve with the use of clever chemistries. Poly l-lactic acid (PLLA) has been shown to increase survival and chondrogenic gene expression of chondrocytes [92,93,94]. Interestingly, PLLA degradation products, such as lactate-based products, can increase chondrogenesis of both natural and OA-compromised chondrocytes [95] Currently, PLLA-based scaffolds are in a period of optimization, with many variables being explored to prevent the production of fibrocartilage in vivo [96]. One such variable explored by Conoscenti et al. [94] was pore size with smaller pore sizes (100 versus 200 μm) improving cell attachment and integration into the scaffold as well as promoting a typical rounded chondrocyte phenotype. Others have considered inclusions to the PLLA material, such as silk fibroin [93] or alginate [96].

## 8. Where Are We with Respect to Growth Factors and Supplements for Cartilage Cell Therapy?

In conjunction with the cell types and the scaffolds, on-going research continues to explore a range of substitutions, or additions, to culture conditions. This is in the hope of optimizing chondrocyte culture and replacing fetal bovine serum (FBS), which has limited availability and wide variability worldwide. Considering this issue, alternate media supplements have been explored, including pooled human platelet lysate and platelet-rich plasma. Platelet lysate does increase proliferation of human adult chondrocytes but at the cost of reduced chondrogenesis [97]. Platelet-rich plasma data are more promising [98,99]. Chondrogenesis was superior, and factors associated with the promotion of cell death were downregulated, thereby promoting a proliferative state. Protein-rich plasma (PRP) also allowed the recovery of OA chondrocytes [99]. This suggests it may be beneficial in combating the inflammatory response associated with surgical intervention and reducing graft failure.

Over the last twenty years or so multiple growth factors have been investigated to improve cell culture conditions [100,101]. These include transforming growth factor-beta (TGF-β) and insulin-like growth factor-1 (IGF-1) both important for promoting chondrogenic fate [102]. TGF-β addition has been shown to recover the loss of chondrogenic properties following monolayer expansion [103]. IGF-1 delivery from a scaffold has been shown to promote GAG content and chondrogenic phenotype for human chondrocytes in vitro [104]. This has also been replicated with BM-MSCs, which increase in cell number upon transfection with IGF-1 [105]. Equine models have confirmed the maintenance of increased collagen II and GAG levels upon transplantation [106]. These grafts are clinically viable, showing significant improvement in gross and histological scoring. This is translated mechanically, with IGF-1-enhanced chondrocytes showing increased stiffness and decreased hydraulic permeability to better replicate the properties of native chondrocytes [107]. This implies that IGF-1-exposed chondrocytes are of better quality and are functionally viable.

## 9. Conclusions

**Figure 4:**
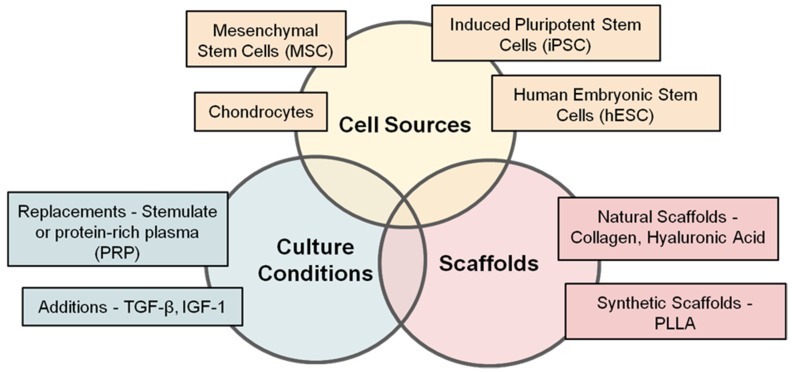
The future direction of cell therapy links together the three fundamental principles of tissue engineering. TGF-β— transforming growth factor-beta, IGF-1— insulin-like growth factor-1, PLLA—poly l-lactic acid.

ACI as a treatment for focal chondral and/or osteochondral lesions has changed very little since its inception, but there remains scope for improvement. The future of ACI now encompasses and embraces the governing principles of tissue engineering (Figure 4). Today, cartilage cell therapy stands on the cusp of another evolution, with a plethora of cell sources, cell types, and biomaterials to develop.

There are many challenges to tackle but probably the hardest remains a long-term surgical treatment for OA joints. Even though ACI is not promoted as a treatment for OA, a recent study suggests that OA chondrons are superior to healthy chondrons in maintaining cell number in early passages and transcriptional activity of key ECM genes [108]. With these cells in mind, there is emerging potential for gene editing via CRISPR-Cas9 [109]. Briefly, this system is encoded with a specific RNA to guide the enzyme Cas9 to a desired target for cleavage. Through the targeted down regulation of matrix metalloprotein-13 (MMP13), generated chondrocytes can increase expression of collagen II [110]. This means gene editing of targets could convey chondrogenic advantages to cells and increase the potential of superior articular cartilage repair. Furthermore, it could be used in OA repair to counter catabolic activity upon re-implantation. The current concern with these techniques is the unforeseen repercussions of gene editing or complications associated with its complexity [111] but they offer further strategies to move the exciting field forward again.

## Figures and Tables

**Table 1:** Surgical treatments to manage articular cartilage injury within the knee. OA—osteoarthritis.

Treatment	Procedure	Pros	Cons	References
Lavage	Washing of the affected joint to clear away cartilage tissue and debris.	Minimally invasive arthroscopic approach, immediate weight bearing.	Only beneficial for acute/early OA, does not encourage repair or regeneration.	[16]
Microfracture	Drilling at the injury site to encourage cell migration to undertake natural repair mechanisms.	Minimally invasive arthroscopic approach, no need to harvest patient tissue.	Only used for lesions <2.5 cm^2^, encourages formation of inferior fibrocartilage, weight bearing limited for six to eight weeks.	[17,18]
Osteochondral allograft transfer	Transfer of a cartilage allograft (sourced from a cadaver or tissue bank) into the patient at the site of injury.	No risk of donor site morbidity and repairs large lesions.	Allogenic tissue (problems with graft availability, cell viability, disease, and immune responses), requires arthrotomy procedure, weight bearing limited for eight weeks.	[19]
Osteochondral autograft transfer (mosaicplasty)	Transfer of a cartilage ‘plug’ from a lower bearing area to the site of injury of the same patient.	Arthroscopic or small arthrotomy approach, aims to produce native hyaline cartilage.	Requires harvesting of healthy cartilage tissue from alternative joint, cannot treat large lesions, problems with tissue integration.	[20]
Osteotomy	Surgical reshaping of the affected joint to remove pressure from the area of cartilage injury.	Delays the need for joint replacement, allows a return to high-impact activity.	Invasive procedure, weight bearing limited for six weeks.	[21,22]
